# A Mobile Gaming Intervention for Persons on Pre-Exposure Prophylaxis: Protocol for Intervention Development and Randomized Controlled Trial

**DOI:** 10.2196/18640

**Published:** 2020-09-14

**Authors:** Laura Whiteley, Elizabeth Olsen, Leandro Mena, Kayla Haubrick, Lacey Craker, Dylan Hershkowitz, Larry K Brown

**Affiliations:** 1 Department of Psychiatry and Human Behavior Warren Alpert Medical School Brown University Providence, RI United States; 2 Department of Population Health Science University of Mississippi Medical Center Jackson, MS United States; 3 Department of Psychiatry Rhode Island Hospital Providence, RI United States

**Keywords:** pre-exposure prophylaxis (PrEP), adherence, mobile gaming intervention, HIV prevention, men who have sex with men (MSM)

## Abstract

**Background:**

In the United States, young minority men who have sex with men (MSM) are the most likely to become infected with HIV. Pre-exposure prophylaxis (PrEP) is an efficacious and promising prevention strategy. However, PrEP’s safety and effectiveness can be greatly compromised by suboptimal adherence to treatment. To maximize the positive impact of PrEP, it is necessary to combine its prescription with cost-effective behavioral interventions that promote adherence and decrease HIV risk behaviors. In this project, we developed a theoretically informed app/gaming intervention to engage young MSM in learning information, practicing behaviors, and improving motivation for HIV preventative behaviors and PrEP adherence.

**Objective:**

The goal of this project was to develop and test a cutting-edge, engaging, and entertaining app/gaming intervention for improving adherence to PrEP and building HIV prevention knowledge, skills, and behavior.

**Methods:**

This study was conducted in two phases. In the developmental phase, we conducted qualitative interviews with young MSM (n=20) to guide the development of the gaming intervention. In the randomized controlled trial, we tested the preliminary efficacy of the gaming intervention compared to a comparison condition among young MSM. Subjects were recruited from the University of Mississippi Medical Center HIV/STI testing clinics (n=60).

**Results:**

Institutional review board approval was received in February 2015. Research activities began in June 2015 and are still ongoing.

**Conclusions:**

This app/gaming intervention aimed to improve PrEP adherence and HIV preventative behaviors in young MSM. Engaging young MSM in learning information, practicing behaviors, and improving motivation for increased adherence to PrEP has the potential to decrease HIV seroconversion. It is important to develop interventions that are enjoyable, engaging, and easily incorporated into clinical settings.

**Trial Registration:**

ClinicalTrials.gov RCT02611362; https://tinyurl.com/y65gkuwr

**International Registered Report Identifier (IRRID):**

DERR1-10.2196/18640

## Introduction

### Background

The primary prevention of HIV infection remains a crucial priority. In 2011, there were 2.5 million new HIV infections worldwide [1]. In the United States, young minority men who have sex with men (MSM) are the most likely to become infected with HIV. Antiretroviral (ARV) medications that reduce the risk of acquiring HIV infection, namely pre-exposure prophylaxis (PrEP), are an efficacious and promising prevention strategy [1,2]. There have been significant advances regarding PrEP including the definitive demonstration that PrEP reduces HIV acquisition, regulatory approval of Truvada (tenofovir/emtricitabine or TDF/FTC) with an indication for sexual HIV prevention, and development of clinical prescribing guidelines. Despite these promising events, the practical implementation of PrEP is challenging [3-10]. Data show that PrEP’s safety and effectiveness could be greatly compromised by suboptimal adherence to treatment, and there is concern about the potential for an increase in HIV risk behavior among PrEP users [11-15]. Due to these challenges, the prescribing of PrEP should be accompanied by behavioral interventions.

Supporting the idea that adherence to medical care is an integral factor in PrEP’s effectiveness, the iPrEx, TDF2, and Partners PrEP clinical trials all conclusively showed that the level of protection from HIV infection depended on how consistently participants took prescribed medication [16-18]. Significantly greater levels of protection occurred among participants with detectable levels of ARVs in these trials. The impact of adherence was also underscored in the FEM-PrEP and VOICE studies, both of which found that few women had detectable levels of ARVs; thus, the studies were unable to demonstrate the efficacy of PrEP [15,19,20]. In 2013, the nested substudy of the Partners trial found that high (>80%) PrEP adherence was associated with 100% efficacy (95% CI 83.7%-100%) [17]. It is clear that the success of PrEP interventions highly depends on good adherence [14-17]. However, concerningly low rates of adherence to PrEP are seen in diverse samples of young Black MSM at high risk of acquiring HIV. Among a sample of 178 young MSM on PrEP (86% identified as Black/African American; median age 26 years; from North Carolina, Washington DC, and California), 36% did not meet the laboratory sensitivity threshold for ≥4 doses/week as measured by FTC/TDF levels in plasma and peripheral blood mononuclear cells at 26 weeks after PrEP initiation [21].

Adherence to PrEP medication is critical; however, engaging patients in comprehensive follow-up care is also imperative [22]. Treatment with PrEP requires consistent contact between patients and clinical providers and includes appointments with clinicians every 3 months for laboratory monitoring, HIV testing, and the detection and treatment of associated side effects [5,9,11-15]. Individuals who are at highest risk of HIV infection often come from populations that historically have been underserved by the health care system [23,24]. Therefore, engaging patients in care could be challenging and will require reinforcement and support for doctors and patients [13,23]. Consequently, behavioral interventions that promote adherence to comprehensive PrEP treatment will need to be tailored to underserved and at-risk populations and will need to reinforce the clinician-patient relationship.

Although there is great optimism about the use of PrEP for HIV prevention, there is concern that PrEP users may take more sexual risks or underutilize traditional risk reduction strategies, such as condom use and HIV and sexually transmitted infection (STI) testing of partners. Behavioral models (behavioral disinhibition and risk compensation) suggest that risk could increase with the reduction of self-imposed constraints or by decreasing individuals’ perceptions of HIV risk. This, in turn, could lead to increased incidence of HIV and other STIs [14,25-28]. Some mathematical and cost-effectiveness models have suggested that even small increases in risk behavior could offset or reverse PrEP’s protective benefits at the population level [13,14]. Data from the iPREX study showed only indirect evidence of increased risk behaviors, as those participants who engaged in unprotected anal sex were more likely to have detectable tenofovir levels than those with less sexual risk taking [17,29]. In real-world clinical settings in which people know that they are actively on a prophylactic medication, behavioral risk taking could measurably increase. Increases in risk behaviors have been documented in the context of microbicide trials, in vaccine trials, and among patients living with HIV on ARV therapy (ART) [30]. Therefore, HIV prevention counseling remains clinically relevant and prudent when prescribing PrEP. This practice is consistent with good clinical care and is recommended in interim guidelines for prescribing PrEP, which state that PrEP has the potential to contribute to effective and safe HIV prevention for MSM if “it is delivered as part of a comprehensive set of prevention services, including risk-reduction and PrEP medication adherence counseling” [22]. Therefore, in order to maximize the positive impact of PrEP, it is necessary to combine the prescription of PrEP with behavioral interventions that promote both adherence and the reduction of HIV risk behaviors [2]. However, these accompanying behavioral interventions need to be cost-effective and easily integrated into clinical settings in which PrEP is prescribed [15]. Additionally, interventions should be enjoyable and tailored to populations targeted for prevention with PrEP. Without these necessary components, integration of behavioral interventions into clinical settings cannot be realistically sustained.

There are many advantages to using newer interactive technology and gaming to promote adherence, rather than traditional face-to-face counseling, including scalability, efficiency, and cost-effectiveness. The use of an intervention that utilizes gaming technology is particularly compelling for use with younger adults who are heavy utilizers of mobile technology and who are most at risk for acquiring HIV. Games can attract and maintain attention, which is a key component for effective behavior change. Compelling, interactive, phone-based games can expose players to essential health-related content countless times and give players unlimited opportunities to rehearse new skills and receive personalized feedback on health choices made within the game [31-33]. Games have been shown to be efficacious in promoting fitness, improving weight management, and improving safer sex skills [31,33-35]. For example, a HIV/AIDS-prevention computer game called *Life Challenge* was developed by the New York State Department of Health to enhance safer sex negotiation by adolescents and young adults. The game showed significant improvement in self-efficacy for partner negotiation and condom skills for those who started with the least self-efficacy [33]. Two pregnancy prevention games, *The Baby Game* and *Romance,* designed for sexually active young adults showed trends in improving knowledge and attitudes about parenting and unprotected sexual behaviors [32].

Video games have also been applied to improve self-management skills and healthy behaviors in those living with asthma, diabetes, and cancer [36-40]. A video game named *Re-Mission,* designed for a wide age range of patients, namely 13-29 years old, with acute leukemia, lymphoma, and soft-tissue sarcoma, showed promising effects as well. *Re-Mission* was designed as an action-adventure game with the main character or protagonist shooting cancer-causing agents in the bloodstream. Players gained points and strength by adhering to medications in the game fantasy world. In a randomized control trial (RCT) with a 3-month follow-up, 375 male and female participants who played *Re-Mission* had significantly improved adherence to trimethoprim-sulfamethoxazole (*P*=.012) and 6-mercaptopurine (*P*=.002) compared to controls after an average of only 10.7 hours of play. Adherence to trimethoprim-sulfamethoxazole was tracked by electronic pill monitoring devices (n=200). The proportion of doses taken correctly by those playing *Re-Mission* was 19% greater than those in the control group. Self-efficacy (*P*=.011) and knowledge (*P*=.035) also increased significantly compared with the control group. Interestingly, the intervention did not affect subjective self-report measures of adherence but did affect the aforementioned objective measures [39,40]. Thus, appealing interactive games can target information, motivation, and skills for medical care and lead to a broad spectrum of desirable outcomes including increases in knowledge, attitude changes, and increased medication adherence [34,35,41-43].

Very few studies describe outcomes of theory-driven PrEP adherence interventions, and there are no other publications on gaming interventions to improve PrEP adherence. Fuchs et al [44] showed that a mobile health intervention using weekly bidirectional messaging (iText) was acceptable and demonstrated promising effects on PrEP adherence in a within-subjects trial design. This sample included MSM from San Francisco and Chicago (one-quarter were under 30 years of age, 13% were Black/African American, 11% were Latino, and 88% completed some college). A pre-post intervention regression discontinuity analysis using clinic-based pill counts showed a 50% reduction in missed doses (95% CI 16%-71%; *P*=.008) and a 77% improvement (95% CI 33%-92%; *P*=.007) when comparing pill counts at quarterly visits before and after iText enrollment. Liu et al [45] reported on the outcome of PrEPmate, a bidirectional text-messaging intervention, on study visit completion and tenofovir diphosphate (TFV-DP) concentrations assessed at 4, 12, 24, and 36 weeks among 121 participants (mean age 24 years; 27% Black, 36% Latino) living in Chicago. Participants who received PrEPmate were more likely to attend study visits (86% PrEPmate vs. 71% non-PrEPmate, OR 2.62, 95% CI 1.24-5.54) and have TFV-DP levels consistent with ≥4 doses/week (72% PrEPmate vs. 57% non-PrEPmate, OR 2.05, 95% CI 1.06–3.94). Although these studies showed promising preliminary effects and feasibility, there are no known definitive, scalable, evidence-based gaming interventions to improve adherence to PrEP.

### Theoretical Framework for Intervention

The Information-Motivation-Behavioral Skills (IMB) model is a well-established conceptualization for improving adherence to treatment as well as decreasing HIV risk behaviors. HIV prevention and ART adherence interventions based on IMB have demonstrated efficacy [46-48], and reviews have suggested that interventions guided by accepted theories of change are more efficacious than those not driven by theory [49]. According to the IMB model, health information, motivation, and behavioral skills are the fundamental determinants of health behavior. In order for a PrEP-related intervention to be successful, the PrEP user must learn information that is directly relevant to PrEP adherence and HIV transmission. Knowledge is a necessary but not sufficient condition for change. Personal motivation to engage in HIV preventative behavior or adhere to treatment regimens (attitudes about health) and social motivation (perceived social and cultural support for performing these acts) are essential for change. Finally, skills for performing adherence behaviors and a sense of self-efficacy must be easily applied to an individual’s cultural and social setting. The most recent review of factors associated with PrEP adherence suggests that adherence can be facilitated by “accurate knowledge of medication benefits,” “medication optimism and self-efficacy for adherence,” and “support provided by peers and providers” [50]. Our intervention addresses these factors within the context of the IMB model. This model, consistent with Social Learning Theory, is broadly applicable and can be used to create theoretically consistent intervention content [51].

### Aims and Objectives

The aims of this project were to develop and test a cutting-edge, engaging, and entertaining app/gaming intervention to improve adherence to PrEP and build HIV prevention knowledge, skills, and behavior. The effectiveness of a novel, scalable, technology driven intervention to improve PrEP adherence is understudied. Consequently, through this RCT, we aimed to leverage technology to engage patients in learning skills, practicing behaviors, and improving motivation for adherence and healthy behaviors. We are not aware of any PrEP-related intervention that utilizes a theoretically informed game. An engaging, technology-based intervention may empower and engage PrEP users, aid over-burdened clinics, and result in improvements in health.

## Methods

### Trial Registration and Institutional Review Board Approval

This research involving human subjects took place at collaborating sites. The protocol was reviewed by the Institutional Review Boards of Brown University, Lifespan/ Miriam/Rhode Island Hospital (RIH), and the University of Mississippi Medical Center (UMMC). This study is also registered on ClinicalTrails.gov (RCT02611362).

### Design

This research was divided into two major phases. First, a developmental phase (n=20, 18-35 years old, receiving PrEP) consisted of formative research to guide the development of the app/gaming intervention. Second, in an RCT phase, we evaluated the preliminary efficacy of the PrEP-related IMB app/gaming intervention compared to a comparison condition — a non-PrEP–related, non-IMB–informed game (n=60, 18-35 years old, receiving PrEP). Subjects were recruited from and consented in person at the UMMC.

### Participants

All MSM initiating PrEP between the ages of 18 and 35 years were eligible for enrollment in each phase of study according to the following criteria: (1) English speaking, (2) initiating PrEP, (3) not enrolled in another PrEP-related study, (4) able to give consent or assent, and (5) not impaired by cognitive or medical limitations as per clinical assessment. Clinical assessment occurred by members of the research team with substantial prior clinical (medical and psychiatric) and research experience in care of young adults and adults. There was no overlap between subjects in the developmental and RCT phases. We enrolled only MSM because they are the group most at risk for acquiring HIV. Limiting the study to MSM allowed for the development of an iPhone app/game that was targeted, acceptable, and engaging for this specific at-risk population. Based on previously acquired data, it was estimated that 65% of participants would be Black/African American, 30% would be Hispanic, and the mean age would be 27 years. A total of 20 participants were recruited for the developmental phase and 60 for the RCT phase over a period of 24 months.

### Description of Intervention Content

The gaming intervention developed for this project by Mission Critical Studios was titled *Viral Combat.* *Viral Combat* was consistent with the IMB Model of Health [52] and promoted increased self-efficacy for adherence to PrEP and HIV preventative behaviors by improving motivation and knowledge about health and prevention and by increasing mastery of skills. Successful games are intuitive, engaging, and inherently rewarding through action and feedback. Many of the attributes of a successful game are a natural fit for a successful IMB-informed intervention.

**Figure 1:**
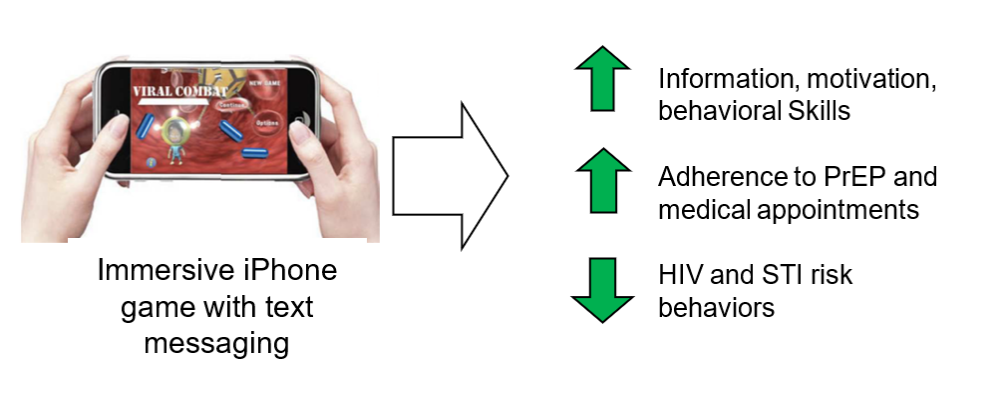
Goals and components of the multilevel gaming intervention. PrEP: pre-exposure prophylaxis; STI: sexually transmitted infection.

Participants playing *Viral Combat* fight off HIV and keep it from entering a virtual body. The game takes place on the surface of the skin, in the arterial system, and in the penile and anal canals. HIV-related educational material (eg, puzzles and quizzes) were tailored to prevent HIV (ie, importance of testing and using condoms) and not tailored to persons living with HIV (ie, how viral load affects transmission and health). Adherence messages were relevant to participants using PrEP for HIV prevention (ie, taking PrEP doses as prescribed corresponds to the level of protection). *Viral Combat* employs graphics, characters, and action content specifically chosen to appeal to our target population. All organ systems are vibrant and distinctive. Answering questions from the doctor or clinician and building adherence and HIV prevention knowledge allows each player to earn strength and points throughout *Viral Combat*. During play, if an answer to a question or skill-building exercise is wrong, the player is alerted, and the correct answer is explained. “Health Facts” reinforce HIV prevention information during scene changes. HIV prevention skills are built with a condom “puzzle” and the “condom use challenge” that promotes continued condom use with all partners. Each level in *Viral Combat* provides new challenges and unique and colorful environments. However, the mission stays the same: Kill the virus and build strength through taking medicine, learn HIV prevention information, improve motivation, and engage with healthy charcters in order to build skills. All character controls and gaming are achieved using touch screen technology on the phone with no additional accessories needed. Throughout the game, the terms “HIV,” “AIDS,” “antiretroviral,” and other identifying verbiage are avoided to protect the players’ medical status and to avoid possible stigmatization that could occur from someone seeing a participant playing the game (See Figure 1).

### Developmental Phase

#### Overview

The goal of the developmental phase (n=20) was to develop and refine the game *Viral Combat* for the intervention. This phase established acceptability and feasibility of the gaming intervention by participants. These steps were accomplished with preliminary work by the scientific investigators in collaboration with Mission Critical Studios. Multiple reviews have demonstrated that behavioral interventions shown to be most efficacious are tailored for the target population and preceded by formative research to inform intervention development [53-55]. During the developmental stage, we elicited feedback from participants regarding the gaming intervention. Necessary changes were made if participants felt the game content or title was revealing, insensitive, or stigmatizing.

#### Individual Qualitative Interviews

In-depth, structured individual interviews about the utility and appeal of the gaming intervention were conducted with approximately 20 participants during the development phase. Interviews continued until redundancy in major themes occurred. We ensured that the assembled intervention was oriented to the appropriate ages, genders, and sexual orientations of participants and that it was relevant and consistent for our target population. Enrollment was monitored to ensure that there was adequate representation of gender, ethnic, racial, sexual orientation, and age diversity among participants. An overview of the IMB Gaming Adherence Intervention and study procedures were presented, and feedback was elicited regarding their relevance. We asked about general reaction to game content (including the title and all screens), and we looked for deeper or more complex emerging themes to guide game and procedure development. For example, if certain game actions were more appealing to participants, then this action style was enhanced or increased. When characters were needed with darker or lighter skin or more or less colorful clothing or gender differentiation, we added these game options. All interviews were audiotaped, and major topics and subtopics from the interviews were coded and reviewed.

Approximately 10 participants were shown the storyboard of the game for approximately 30 minutes; qualitative feedback was elicited, and game development for the iPhone occurred. After the development of the iPhone version of *Viral Combat*, 9 of the 20 participants played the game. These 9 participants were shown each of the game levels on the phone by the interviewer and were then able to play each level on their own. After playing the game, these 9 participants completed qualitative interviews, and written or quantitative feedback was obtained. Quantitative feedback was collected from these participants using versions of the Client Service Questionnaire (CSQ-8) [56] and Session Evaluation Form (SEF) [57]. Both of these instruments were developed to measure client satisfaction and perspectives on intervention aspects. Responses to items on these questionnaires helped determine the initial feasibility and acceptability of the intervention. If the gaming intervention consistently received low CSQ-8 or SEF scores (<24 on the CSQ-8 and <30 on the SEF) it was modified until scoring on these measures improved above stated thresholds. Based on these data, *Viral Combat* was modified by Mission Critical Studios to create a final version of the multilevel gaming intervention for the RCT.

### Randomized Controlled Trial

#### Overview

During the RCT phase, we evaluated the preliminary impact of the gaming intervention compared to a comparison group (iPhone with non-PrEP comparison game) using a parallel design with 60 participants on PrEP. Recruitment, enrollment, and implementation of the intervention were conducted in a rolling manner. Randomization (1:1) occurred after enrollment using a computerized, random assignment program. Research staff performed randomization, so the allocation sequence was unknown to the participants and UMMC staff conducting the recruiting. However, the trial was not blinded.

Each participant in the RCT received an iPhone with a paid data service plan for the study duration (iPhone 4S; US $100; US $60/month data package through Lifespan/AT&T). Participants in both arms were given phones because at the time of study commencement, iPhone ownership was not ubiquitous in this clinical population. Participants were able to keep the phone at the end of the study, but the data service plan was not continued. Both the intervention and control games were downloaded at no cost from the App Store onto the iPhones provided to participants at time of enrollment. Participants, therefore, could access the game from any location, at any time. There were no prompts nor reminders sent to participants to play the game. Neither game allowed for communication between participants and study staff. Study staff members were not blinded to condition in the RCT phase of the study. Study staff had limited contact with participants during RCT assessments, as these were done online through REDCap.

#### Viral Combat Gaming Intervention

**Figure 2:**
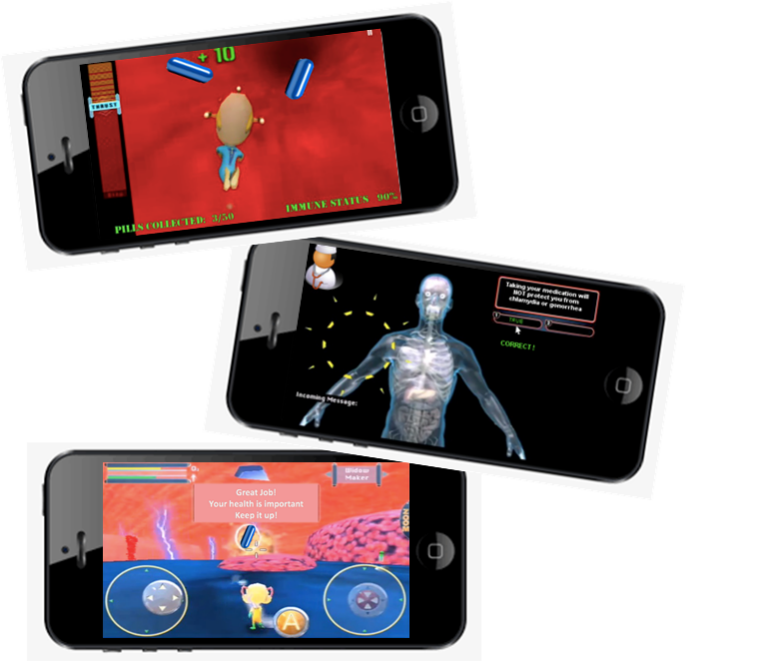
Images from the app/game Viral Combat.

The gaming intervention, shown in Figure 2, was designed to improve information, motivation, and skills regarding adherence and HIV preventative behaviors throughout play. All content was chosen for its appropriateness for the target population with refinements made during the developmental phase. With input from the research team and PrEP users, we produced a sensitive, stylistically and theoretically consistent intervention. Each level in *Viral Combat* provides new challenges and unique and colorful environments. Throughout the gaming intervention, subjects continued routine clinical care visits and HIV testing (as recommended by PrEP prescribing guidelines) in the PrEP clinic.

#### Comparison Condition (Non-IMB iPhone Game)

The comparison condition was matched with the multilevel gaming intervention for appeal, time, and attention. Subjects in the comparison condition received smartphones with the same data service plan as the intervention arm. Smartphones given to participants in the comparison condition had a stylistically similar non-PrEP, non-IMB game designed by the same company, Mission Critical Studios [58]. This was the same game from which *Viral Combat* was adapted. This similarity was important as qualitative assessments did not assess participants’ reactions to the control game. The iPhone game in the comparison condition had a look and feel that was very similar to the intervention game but lacked IMB-related, PrEP-related, and HIV prevention–related content in order to control for attention, time, and any influence the receipt of a smartphone or game had on adherence behaviors or the physician-patient relationship. Notably, participants in the comparison condition could hear from those in the IMB intervention about the IMB app/game, but they did not have access to it on their phone. Although it was possible that the participants in the comparison condition could play the game once on an intervention participant’s smartphone, it was unlikely to be a frequent occurrence. Nevertheless, we assessed familiarity and usage of the game by self-report at follow-up. In addition, we assessed the occurrence of cross talk between subjects in the two conditions at all follow-ups by asking those in the intervention condition, “Have you spoken to anyone about the game you played in the study (yes or no)?” and for those in the comparison condition, “Have you spoken to anyone about a game related to PrEP (yes or no)?”

#### Measures and Assessments

All assessments took place in a quiet, private room near or in the PrEP clinic. Assessments occurred at baseline, 12 weeks post-enrollment, and 24 weeks post-enrollment and took 45 minutes to complete.

An audio-assisted computer self-interview using REDCap was used to assess behavior since it was confidential, allowed for complex branching and skip patterns, and detected greater rates of risk behavior [59]. Standard instruments were administered to gather demographic data (including age, level of education, sexual orientation, socioeconomic status, race, ethnicity and stability of housing). The measures listed in Table 1 were used to evaluate HIV-related and STI-related knowledge, attitudes, and risk behaviors.

**Table 1:** Linkage between constructs, intervention foci, and assessment instruments.

IMB^a^ constructs	Intervention foci	Assessment instruments
Information: HIV and STI^b^ sexual risk knowledge	HIV knowledge, perceived vulnerability to HIV/STIs	HIV/STI knowledge scales
Motivation: adherence and risk attitudes, intentions, and self-efficacy	Motivation for adherence, motivation for safer sex	Self-efficacy for adherence and sexual safety, adherence and risk attitudes
Behavior: medical adherence, sexual risk, and substance use	Decrease in perceived barriers, increased medication and visit adherence, safer sex skills	Adherence and sexual risk self-report, ARV^c^ levels

^a^IMB: Information-Motivation-Behavioral Skills.

^b^STI: sexually transmitted infection.

^c^ARV: antiretroviral.

#### Primary Outcomes

The primary outcomes included intracellular levels of TFV-DP and emtricitabine (FTC-TP) in red blood cells, as measured using dried blood spots. TFV-DP levels provided a measure of long-term adherence over the preceding month (like hemoglobin A1C), and a detectable FTC-TP level provided information about recent dosing (ie, if FTC-TP was detectable, a recent dose was ingested). The level of intracellular TFV-DP was used to estimate how many doses per week the participant took on average (eg, 7 doses per week, 4-7 doses per week, 2-4 doses per week, <2 doses per week). To collect TFV-DP and FTC-TP levels, 25 µL of blood was drawn from a finger prick.

#### Secondary Outcomes

The secondary outcomes described in this section were collected from self-report using online questionnaires or from abstraction from medical records. Changes in self-reported measures were evaluated at 12 weeks and 24 weeks.

Self-report of PrEP adherence consisted of the following 3 items developed by Wilson et al [60] that ask about adherence in the past month: (1) “On average, how would you rate your ability to take PrEP as your doctor prescribed?” (2) “How often did you take PrEP as your doctor prescribed?” and (3) “What percent of the time were you able to take PrEP as your doctor prescribed?”

To collect medical history at both follow-up assessments, research staff at the STI/HIV testing clinic abstracted from the electronic medical records, with participant consent, the number of PrEP-related medical visits kept and missed in the past 12 weeks.

The Risk Behavior Assessment is a reliable and valid computer-assisted structured interview used to assess self-reported sexual behaviors. It assesses types of sexual behavior (ie, anal, oral, vaginal) in the past 12 weeks, frequency of sex, and number and gender of partners. Additional questions cover condom use, sex with high-risk partners, frequency and quantity of substance use, and having sex while using alcohol and drugs [61].

To assess HIV knowledge, we used the HIV Knowledge Scale, which assesses knowledge about issues such as risks for HIV using 18 items with “true,” “false,” or “do not know” as response options. Test-retest reliability (r=0.73) and internal consistency (reliability coefficient=0.90) were satisfactory in studies with at-risk young adults [62]. To assess STI knowledge, we used the STI Knowledge Questionnaire, which uses similar response options for 10 items that assess risk for and treatment of STIs. Test-retest reliability (r=0.88) and internal consistency (reliability coefficient=0.86) were both satisfactory in studies with at-risk young adults [63].

The Attitudes towards PrEP Adherence checklist originated from attitude items used in several AIDS clinical trials groups and was modified to reflect adherence to PrEP. The checklist assessed 16 common barriers and 10 facilitators to taking ARV as prescribed.

Motivational readiness for adherence to PrEP and PrEP-related medical visits was assessed using Rollnick’s Readiness Ruler [64]. Respondents rated how ready they were to take PrEP as prescribed and to go to PrEP-related medical appointments on a scale of 1 (not ready) to 10 (ready to be consistent or already consistent). Subjects also completed the 10-item Likert-style IMB PrEP Motivation Scale from the LifeWindows Project Team. It was modified to assess personal and social motivations for PrEP, rather than ART [65].

The PrEP and appointment self-efficacy measure was developed based on Bandura’s theory of self-efficacy [51] and was shown to have strong reliability (reliability coefficient >0.8) in a study of HIV medication adherence [66]. The instrument consists of Likert-style items (with 5 response options): 3 items assessed self-efficacy for taking PrEP as prescribed, and 3 items assessed self-efficacy for attending PrEP-related medical appointments. The IMB PrEP Behavioral Skills Scale has 14 Likert style items and was modified to assess perception of the ability to perform necessary PrEP skills rather than ART skills. It has an internal consistency of 0.9 when use with infected adults [65].

#### Moderators

In addition to demographic factors, several psychosocial factors may influence adherence, so the project assessed these factors for exploratory analyses to further characterize the sample. Some factors, such as the quality of the relationship with health care providers could function as a moderator or could be influenced by the intervention.

The 5-item relationship with providers measure assessed the perceived relationship with health care providers using Likert-type items suggested by an ART adherence intervention with adults (Project HEART) [67].

The 6-item social support for medication adherence measure assessed social support for taking medications, going to medical appointments, and other tasks related to adherence using Likert-style items with a 4-point scale. It is being used in AIDS clinical trials. A single score for social support can be generated from these items, or single items can be analyzed, such as support for medical appointments [68].

Mental health issues were assessed using the Brief Symptom Inventory, which requires only 8-10 minutes to complete. It yields 9 primary symptom scales and global indices and has norms for adolescents and adults. The reliability, validity, and utility of the Brief Symptom Inventory instrument have been tested in more than 400 research studies. Internal consistency for the subscales (dimensions) ranges from 0.71 to 0.85 [69].

The Alcohol, Smoking and Substance Involvement Screening Test (ASSIST V2.0) is an 8-item questionnaire that screens for all levels of problem substance use. The instrument covers tobacco, alcohol, cannabis, cocaine, amphetamine-type stimulants (including ecstasy), inhalants, sedatives, hallucinogens, opioids, and “other drugs.” The ASSIST V2.0 demonstrates significant concurrent, construct, predictive, and discriminative validity. The ASSIST scores are comparable with other measures of substance use and are able to discriminate between low-risk, moderate-risk, and high-risk use [70].

### Statistical Analysis

#### Hypothesis One

The multilevel gaming intervention was judged by participants to be feasible, appealing, relevant, and useful. A total of 20 qualitative interviews during the developmental phase assessed acceptability and appeal of the intervention. We reviewed the interviews for any game material that was unfavorable to participants and made revisions. Any game modules that received a mean score <20 points on the SEF (smaller scores indicate less satisfaction) were reviewed to determine what content should be revised, changed, or discarded.

#### Hypothesis Two

Compared to subjects in the control group, participants in the multilevel gaming intervention are anticipated to show improved adherence, improvements in biological measures (higher blood levels of TFV-DP and FTC-TP), decreased HIV risk behaviors, greater increases in HIV knowledge, and improved self-efficacy and attitudes for treatment adherence compared to those in the comparison condition. Generalized estimation equations with a Poisson distribution and log link function will be used to model outcomes with a count distribution (self-report of adherence, unprotected sex, medical appointments). The generalized estimation equation analysis will account for nesting of assessments within participants and allows for data that follow nonnormal distributions. The IMB constructs and levels of TFV-DP are continuous variables and will be evaluated using repeated measures analysis of variance. FTC-TP levels reveal a recent ingestion (yes/no) and will be analyzed by tests of proportional difference. In all analyses, we will test for differences in linear change over time between intervention and control groups on each outcome variable. We will examine baseline differences across all outcome measures to assure that randomization was successful and that groups were equivalent at baseline. We will control for demographic variables that show pretest differences. Hierarchical linear modeling analyses are robust in cases of few missing data. However, if there is a large amount of missing data, last observation carried forward techniques will be employed to deal with attrition and missing values. Outcomes like attendance at a PrEP services appointment can be extracted from medical records, regardless of subject attrition.

#### Power

As this is an intervention development study and the impact of the experimental intervention is not known, there may not be adequate power to determine the efficacy of the multilevel gaming intervention. Pilot studies are not designed to provide accurate estimates of effect sizes upon which to base large trials [71]. Nevertheless, the pilot RCT may provide a “signal” of impact on outcomes and IMB constructs. Power was estimated using Monte Carlo simulation in Mplus 7.11. We assume 85% retention over the trial period. For the primary outcome, assuming an initial rate of 16 doses per month, this study will have a power of 0.80 to detect a rate ratio of 1.28. In other words, this study will be able to detect an increase from 16 to 20 doses per month (one additional dose each week), assuming the control group maintains 16 doses per month. For the repeated measures analyses of variance, this study will have power to detect a large effect size (Cohen *d*=0.80).

### Incentives

Subjects were reimbursed US $50 for participating in a qualitative interview during the developmental phase. During the qualitative interview phase, participants were also reimbursed US $5 for every level of the game they completed, up to US $30. During the RCT phase, subjects were reimbursed US $50 for baseline, 12-week, and 24-week assessments. Each participant in the RCT received an iPhone with a paid data service plan for the study duration (iPhone 4S; US $100; US $60/month data package through Lifespan/AT&T). Participants in both arms were given phones, because at the time of study commencement, iPhone ownership was not ubiquitous in this clinical population.

### Ethical Considerations

To protect participants’ confidentiality, the following measures were taken. Participants’ research data were identified using a numeric ID only. Any records containing potentially identifying information were kept secure and separate from other research data. All physical records were kept in a locked file, and electronic data were password-protected. Study materials were only accessible to research staff. Data collection took place in a secure and supervised clinical setting or with Health Insurance Portability and Accountability Act-compliant software (REDCap). All study personnel completed training in Human Subjects Research Protection and Health Insurance Portability and Accountability Act regulations (Collaborative Institutional Training Initiative Program) and received certification. These certifications were kept current in compliance with hospital policies.

Engagement in game analyses, provided by Mission Critical Studios, was participant nonspecific and generalized. For example, Mission Critical Studios collected data on game usage like any other app or website owner that shows percentage of times players of the game complete levels (eg, 50% of players completed level 5). These data were not linked to a particular participant or phone in use. The phone was designated as owned by Lifespan (the organization affiliated with the study) as opposed to the study participant. The gaming data were not connected nor linked to any participant’s name, demographic information, or health status.

To further protect the privacy of the study participants, we obtained a Certificate of Confidentiality from the US Department of Health and Human Services. With this certificate in place, the researchers cannot be forced to turn over identifying information about a study participant in any federal, state, or local criminal, administrative, legislative, or other proceedings. This certificate does not prevent a study participant from volunteering to turn over their research information nor does it prevent researchers from providing research-related information to others when requested by the study participant.

## Results

This is an ongoing study. Approval was obtained from the affiliated institutional review board in February 2015. Research activities began in June 2015 and are still ongoing. Therefore, we cannot yet report on the effect of the intervention on the desired outcomes. Results will be available at a later date.

## Discussion

### Review

The use of ARV medications, such as PrEP, to reduce the risk of acquiring HIV infection is a promising prevention strategy. Optimal PrEP treatment requires simultaneous medical care and behavioral interventions to promote adherence and safer sexual behaviors. For interventions to be successful, they must be targeted to populations most at risk, as well as engaging and theory-driven. If *Viral Combat* proves to be effective in future trials, the intervention would be easy to scale up to larger clinical settings.

### Limitations

This study had limitations. The trial was unblinded; participants were aware of their study arm assignment, which could influence how they reported on outcomes of interest. Time spent gaming was collected by self-report, and individualized paradata were not collected. Therefore, individual participant’s playing time and engagement with the game were not verified. Also, the sample size was small and recruited from the same urban area of the South; therefore, findings may not be generalizable to MSM in other locations.

### Conclusion

There are many advantages to using newer interactive technology, such as mobile gaming and text messages, rather than traditional face-to-face counseling, including scalability, efficiency, cost-effectiveness, and appeal. Given the significant health sequelae associated with HIV infections and the paucity of data on PrEP-related adherence and behavioral intervention programs, the knowledge to be gained from this research is significant.

## Appendix

Multimedia Appendix 1 NIH Peer review summary statement.

Multimedia Appendix 2 CONSORT EHEALTH checklist (v 1.6.1).

## Abbreviations

ART: antiretroviral treatment
ARV: antiretroviral
CSQ-8: Client Satisfaction Questionnaire
FTC-TP: emtricitabine
IMB: Information-Motivation-Behavioral Skills
MSM: men who have sex with men
PrEP: pre-exposure prophylaxis
RCT: randomized controlled trial
SEF: Session Evaluation Form
STI: sexually transmitted infection
TFV-DP: tenofovir diphosphate
UMMC: University of Mississippi Medical Center
