# Supplementary material for: A Mobile Gaming Intervention for Persons on Pre-Exposure Prophylaxis: Protocol for Intervention Development and Randomized Controlled Trial
Source: JMIR Res Protoc. 2020 Sep 14;9(9):e18640. doi: 10.2196/18640 (PMC7522735; doi:10.2196/18640)
Supplement: Multimedia Appendix 1 [file resprot_v9i9e18640_app1.pdf]

SUMMARY STATEMENT
(Privileged Communication)

PROGRAM CONTACT:
Susannah Kamath Ph.D.
301-443-3952
allisonsu@mail.nih.gov

Release Date: **08/06/2014**

Application Number: **1 R34 MH104068-01A1**

Principal Investigator

WHITELEY, LAURA B MD

Applicant Organization: RHODE ISLAND HOSPITAL

Review Group: **ZMH1 ERB-K (07)**
National Institute of Mental Health Special Emphasis Panel
NIMH HIV/AIDS Review

Meeting Date: **07/10/2014**
Council: **OCT 2014**
Requested Start: **09/01/2014**

RFA/PA: **PAR12-279**
PCC: **9A-ASPA**

Dual IC(s): **AI**

Project Title: **A Multilevel Gaming Intervention for Persons on PrEP**

SRG Action: **Impact Score: 28**
Next Steps: **Visit http://grants.nih.gov/grants/next_steps.htm**
Human Subjects: 30-Human subjects involved - Certified, no SRG concerns
Animal Subjects: 10-No live vertebrate animals involved for competing appl.
Gender: **3A-Only men, scientifically acceptable**
Minority: **1A-Minorities and non-minorities, scientifically acceptable**
Children: **1A-Both Children and Adults, scientifically acceptable**
Clinical Research - not NIH-defined Phase III Trial

Project Year	Direct Costs Requested	Estimated Total Cost
1	150,000	238,500
2	150,000	238,500
3	150,000	238,500
TOTAL	450,000	715,500

ADMINISTRATIVE BUDGET NOTE: The budget shown is the requested budget and has not been adjusted to reflect any recommendations made by reviewers. If an award is planned, the costs will be calculated by Institute grants management staff based on the recommendations outlined below in the COMMITTEE BUDGET RECOMMENDATIONS section.

1R34MH104068-01A1 WHITELEY, LAURA

RESUME AND SUMMARY OF DISCUSSION: This R34 resubmission proposes to test the feasibility, acceptability and preliminary efficacy of a PrEP (Pre-exposure Prophylaxis) adherence intervention using multi-level technology that integrates a medication monitoring device (Wisepill) with an interactive smartphone based app/game that is engaging for young adult MSMs and promotes adherence to PrEP. The study incorporates the Information-Motivation-Behavioral Skills (IMB) Model into the development of the gaming content. Strengths include the focus on PrEP use and young adult MSM, a population at high risk for HIV acquisition, along with the use of the IMB theoretical-framework. The applicant, an early career investigator with solid technology and adherence trials, has assembled a well-qualified team supported by an excellent research environment. The resubmission is highly responsive to the previous reviews; the application is strengthened by the increased sample size for the pilot RCT, additional IBM expertise, strong fidelity monitoring, and clear recruitment plans. Nonetheless, weaknesses were identified, such as limited description of the adaptation process and content tailoring. Specifically, reviewers questioned the feasibility and acceptability of the revised game content for HIV-young adults, along with a request for more details about the content adaptation activities in the formative phase. Other minor weaknesses included limited information about RCT measurement, minimal description of the control condition, feasibility of recruitment, and concerns about sufficiency of the sample size, and the subsequent stratification and effect size justification. Although data aggregation was purported, the use of an external gaming company generated questions about data security and data ownership. The foci of this application are highly significant, and the investigative team is well-qualified to execute the study. However, several minor weaknesses were presented at this time.

DESCRIPTION (provided by applicant): The use of antiretroviral medications to reduce the risk of acquiring HIV infection (Pre-exposure Prophylaxis, PrEP) is an efficacious and promising new prevention strategy. In published studies, failure of PrEP was associated with poor adherence and low plasma drug levels. This indicates that optimal PrEP treatment will require behavioral interventions to promote adherence. Averting increases in risk behavior will also be essential for maintaining PrEP's protective effects. Interventions will need to address on-going HIV risk behavior and be relevant for persons using PrEP (e.g. young adult MSM). Borrowing from recent literature in HIV prevention and treatment adherence, interventions that utilize intuitive easy to use technologies with motivational components show promise for behavior change and dissemination. This study will examine a multi-level technology that integrates a medication monitoring device (Wisepill dispenser) with an interactive smartphone based app/game that is immersive and appealing. This novel, but intuitive multi-level technology will measure PrEP adherence, promote engagement in treatment and safe sexual behavior. The smartphone app/game will be developed to include content consistent with the Information-Motivation-Behavioral Skills (IMB) Model. While gaming, participants will experience absorbing action-oriented adventures that increase information about their health (e.g. knowledge about PrEP treatment and HIV), improve motivation (e.g. action-figures experience health benefits of adherence), and build skills (e.g. utilize clinicians as partners, condom self efficacy). Adherence data from participants' Wisepill device will be integrated into the app/game wirelessly and will translate into enhancements during game play. In-depth interviews with young adults on PrEP and an open trial of the intervention will inform the development of the intervention and procedures. A small randomized controlled pilot study among 50 participants on PrEP will examine the preliminary efficacy of the intervention (integration of the Wisepill dispenser with the IMB informed app/game). It is hypothesized that, compared to subjects in the control group, participants in the IMB Gaming Intervention will show: improved adherence to PrEP, higher blood ARV levels, decreased HIV risk behaviors, and improved self efficacy and attitudes for PrEP treatment adherence.

PUBLIC HEALTH RELEVANCE: This study will develop and test a novel, smartphone based gaming intervention to improve adherence to medication to prevent HIV (known as Pre-exposure Prophylaxis, PrEP) and to decrease HIV risk behaviors among men who have sex with men (MSM). In the intervention, participants will engage with an immersive app/game on their iPhone that is connected to smart, electronic pill organizer/dispenser. Data on adherence from the pill dispenser will be integrated into the app/game wirelessly and will translate into enhancements during game play. While gaming, participants will gain information about their health, improve motivation for PrEP and medical appointment adherence, and practice healthy behaviors. If the intervention is found to be effective, it can be tested in a larger study and then disseminated to other people taking PrEP.

CRITIQUE 1:

Significance: 1
Investigator(s): 1
Innovation: 2
Approach: 2
Environment: 1

Overall Impact:
This application is a revised grant submission, effectively addressing the majority of previously raised concerns. Sample size for the developmental and pilot RCT phase have been adjusted (i.e. increased), expertise for the use of the IMB framework has been added, intervention fidelity is monitored by more objective measures, and clarity has been provided regarding the recruitment of participants and focus on young MSMs only as the target population for this intervention. In summary, the investigators are proposing to test the feasibility, acceptability and preliminary efficacy of a PrEP adherence intervention using multi-level technology that integrates a medication monitoring device (Wisepill) with an interactive smartphone based app/game that is immersive and appealing to young MSMs and promotes adherence to PrEP. This application addresses a critical problem in a population at high-risk for HIV acquisition. Led by an early career investigator and supported by senior investigators with expertise in the population, intervention development, use of gaming technology, and implementation of PrEP studies has potential to improve our understanding of the utility of gaming technologies to improve adherence to PrEP among populations at high-risk. Overall this application, grounded in the Information-Motivation-Behavioral Skills model, shows promise as a stepping stone towards a larger clinical trial.

1. Significance:
Strengths
- Addresses the critical issue of PrEP adherence in adolescent and young MSM at risk for HIV acquisition.
- Focuses on adapting an existing HIV adherence gaming intervention used with HIV infected adolescents to increase adherence to PrEP among this population.
- Gaming technology has the potential for widespread dissemination of intervention and is appealing to young adults.
- The integration of a behavioral intervention component into a PrEP adherence intervention will address potential risk compensation as has been associated with PrEP use.

Weaknesses
- [None noted]

2. Investigator(s):
Strengths
- This is a well-qualified group of researchers with expertise across the essential elements of the

study including exploratory and experimental methods; intervention development/adaptation; feasibility and acceptability testing; application of gaming technology; implementation of PrEP trials; and young populations at risk for HIV.

- Dr. Whiteley is an early career investigator with an interest of technology and its applications to reduce the burden of HIV in high-risk populations.
- Dr. Brown is a senior investigator with experience in areas of HIV risk and the efficacy of HIV prevention programs.
- Dr. Chan is the Director of the HIV/STI and PrEP Clinic and currently funded through a K23 award. No salary is requested for his efforts.
- Dr. Lally is a senior investigator with experience related to HIV prevention research that includes biomedical interventions and evaluations. Dr. Fisher has been added as a consultant for greater IMB expertise. He is the developer of the IMB model and an expert in its use for adherence and risk behavior.

Weaknesses

- [None noted].

3. Innovation:

Strengths

- Delivery of the intervention via gaming technology is the most exciting part of this application. Combining it with real-time adherence technology adds to the enthusiasm for this project. It potentially addresses many barriers associated with the dissemination of HIV prevention programs.
- The researchers build on a gaming intervention that has already been developed and tested in the context of HIV medication adherence.
- The development of a theoretically-informed gaming intervention that aims to enhance information about HIV, motivation and behavioral skills.

Weaknesses

- [None noted]

4. Approach:

Strengths

- Careful thought has been given to the processes for adapting and piloting the intervention.
- The inclusion of a time-matched control arm in the pilot RCT is a strength.
- The iterative adaptation of the intervention with participants from the target population in an open trial preceding the RCT allows for maximum opportunity to modify the intervention within the given time frame.
- The use of a real-time monitoring advice to measure PrEP adherence will add to the body of literature on adherence measurement technologies.
- Game usage (dosage of intervention) is measured by self-report and data collected by gaming developing company. This approach increases monitoring of intervention fidelity, providing objective measures regarding dosage and frequency of intervention.
- Tailoring the adaptation of the intervention to the needs of young MSMs, who are at high risk for HIV acquisition, is a strength of this application.
- Details regarding recruitment of participants including the recruitment pool are adequate and recruitment goals seem to be feasible.

Weaknesses

- More detail could have been provided regarding the assessment of the main outcome of the intervention (i.e. adherence to PrEP as measured by the Wisepill). Baseline adherence data likely would not be available given the current research design.

5. Environment:

Strengths

- The collaboration between university, clinical and businesses (i.e. gaming developer) contributes to the ability of the research team to achieve the objectives of this application.
- Great support letters from clinical and business partners.

Weaknesses

- [None noted]

Protections for Human Subjects:
Acceptable Risks and/or Adequate Protections
Data and Safety Monitoring Plan (Applicable for Clinical Trials Only):

- Acceptable

Inclusion of Women, Minorities and Children and not IRB Exemption #4.

- Sex/Gender: Distribution justified scientifically
- Race/Ethnicity: Distribution justified scientifically
- Inclusion/Exclusion of Children under 21: Including ages < 21 justified scientifically

Resubmission:

- Resubmission:

Resource Sharing Plans:

- Unacceptable
- No resource sharing plan was provided for this application.

Budget and Period of Support:
Recommend as Requested

CRITIQUE 2:

Significance: 2
Investigator(s): 1
Innovation: 3
Approach: 4
Environment: 1

Overall Impact:
Suboptimal adherence to PrEP reduces efficacy to prevent sexual HIV transmission in HIV-MSM. In this application, PrEP adherence is promoted by integrating a medication monitoring device (Wisepill) with an iPhone smartphone app/game. PrEP adherence and safer sex behaviors are outcomes that will be measured by opening the pill cap (sends wireless signal to gaming software), higher serum ARV level, lower risk behavior, and higher self efficacy for PrEP adherence compared to a control.
This application is, for the most part, responsive to prior concerns. Dr. Whiteley has increased her effort to 25%, the study population is targeting MSM with 65% African Americans, the sample size for the RCT is increased RCT, TFV/FTC serum levels will be analyzed, measures of adherence are extended to 24 weeks (from 12), and there is an attention/ time control. This gaming app is developed for iPhones, although can be adapted for Android OS. Literature indicates low usage of smartphone gaming apps. IMB addresses rational concerns whereas, adherence and risk behavior are influenced by emotion, relationship pressures, substance use and sensation seeking. These are not sufficiently addressed in the application.

1. Significance:
Strengths
- Integrates a medication monitoring device (Wisepill dispenser) with an interactive smartphone based app/game to enhance PrEP adherence, promote engagement in treatment, and decrease HIV risk behaviors. Adherence data from participants' smart cap will be integrated into the app/game wirelessly.
- In-depth interviews with young adults attending a PrEP clinic, and an open trial of the intervention will inform development.
- Participants in the IMB Gaming Intervention are hypothesized to demonstrate improved adherence to PrEP, higher blood ARV levels, decreased HIV risk behaviors, and improved self efficacy and attitudes for PrEP treatment adherence at 12 weeks post-intervention:

Weaknesses
- Age and cultural appropriateness remain inadequately described.

2. Investigator(s):
Strengths
- Dr. Whitely completed a fellowship T32 training in Child & Adolescent Bio behavioral HIV research. Dr. Whiteley and Dr. Brown co-Direct Young Adult Behavioral Health Program at Rhode Island Hospital.
- The PrEP clinic is directed by Dr Chan.
- CFAR Prevention Core is directed by Drs. Brown and Lally. Dr. Lally led PrEP studies with adolescents (ATN 110/113).
- Dr. Anderson brings expertise on Tenofovir.
- Mission Critical Studios is an experienced game development team.

Weaknesses
- [None noted]

3. Innovation:
Strengths
- Integrates medication dispenser to measure opening pillbox with iPhone following IMB model for an adapted interactive game.

Weaknesses
- “Viral Combat” will be adapted from an ongoing pilot gaming adherence program for HIV+ youth, somewhat diminishing innovation.
- Tailoring is a strength of gaming as described in the application, but where is the tailoring in this project?

4. Approach:
Strengths
- Qualitative interviews with 20 MSM (mean age 27) who are on PrEP to review the adapted game. An open trial of 20 will also review and provide feedback. During the first month, participants will receive Wisepill alone. Then, the smartphone IMB Gaming Intervention will be added to give time to adapt to Wisepill.
- 24-week randomized controlled pilot study (n=50) to test the preliminary efficacy of the IMB Gaming Intervention that integrates the electronic medication dispenser and the IMB informed app/game compared to a time, attention, and technology matched group to evaluate the IMB Gaming Intervention in improving treatment adherence (measured by medication dispenser openings, clinic visits attended, self-report) and biological measures (ARV levels) and HIV risk behaviors
- Consistent with the IMB model, the intervention will emphasize information about their health (e.g. knowledge about PrEP treatment and HIV/STIs), improve motivation (e.g. action-figures

experience health benefits of adherence), and builds skills (e.g. utilize clinicians as partners, condom self-efficacy).

- Primary outcome will be the Wisepill openings measured daily for 6 months. Secondary outcomes self report of medication adherence and sexual risk, HIV related knowledge and attitudes, clinical records (appointments kept, STI treatment), and ARV levels at 12 weeks and 24 weeks.
- Missed doses trigger Wisepill to send data to a database read by mission Critical software; sends a graphic reminder to the phone as developed in an ongoing R01 trial.
- A similar gaming program was highly rated by HIV+ MSM in an ongoing trial by the team

Weaknesses

- It appears that the qualitative interviews are for the purposes of acceptability and not to develop storylines.
- “Viral Combat” will be adapted and refined from the Mission Critical Studios game “Dr. Nano X: Incredible Voyage Inside The Body” that was created for a younger population. How will this be relevant to the current population?
- Mission Critical collects aggregate data on time spent on game, % at each level. Who has control of these data? Will these data be used outside this study?
- The technology attention time control is not described. If not as interesting, may have higher attrition in the control group.

5. Environment:

Strengths

- Conducting a NICHD-funded study to develop a mobile gaming app to improve adherence for youth and young adults living with HIV and is able to recruit HIV+ young adults from the Miriam Hospital Immunology Clinic, the site of the PrEP clinic for this project.
- The population is HIV- MSM, attending a PrEP clinic so it should be feasible to reach recruitment targets.

Weaknesses

- [None noted]

Protections for Human Subjects:

Acceptable Risks and/or Adequate Protections

- Only pill opening events, designated by a Wisepill ID number, are recorded in Wisepill database.

Data and Safety Monitoring Plan (Applicable for Clinical Trials Only):

- Acceptable
- DSMP well described.

Inclusion of Women, Minorities and Children and not IRB Exemption #4.

- Sex/Gender: Distribution justified scientifically
- Race/Ethnicity: Distribution justified scientifically
- Inclusion/Exclusion of Children under 21: Including ages < 21 justified scientifically
- Children 18-21 included.

Biohazards:

- Acceptable
- Venipuncture only.

Resubmission:

- This application demonstrates responsiveness to most of the concerns from previous submission.
- Viral Combat will be adapted and refined from the Mission Critical Studios game Dr. Nano X: Incredible Voyage Inside the Body that was created for a younger population. How will this be relevant to the current population?

Budget and Period of Support:
Recommend as Requested

CRITIQUE 3:

Significance: 4
Investigator(s): 2
Innovation: 2
Approach: 4
Environment: 1

Overall Impact:
Overall, the proposed gaming approach is well justified for adherence to PrEP. It is the case, however, that this application largely ignores the aim of fostering safer sex through this game. Indeed, the Approach does little to describe how this will be achieved. Throughout the application, it appears that the safer sex aim is included with very little thought as to how it will be achieved. This is highly problematic given the thoughtful review in this application of the issues relative to risk compensation for person taking PrEP. The primary aim (adherence) is significant and the plans for achieving this aim are largely strong.

1. Significance:
Strengths
- The need for a gaming approach to PrEP adherence and to avoid PrEP-related risk compensation is clear in this application.
- The selection of minority MSM for this pilot study is well justified.

Weaknesses
- Although evidence reviewed does indeed support the use of gaming for adherence, none of the evidence reviewed fully addressed the potential for gaming to increase safer sex behaviors. The gaming plan may work well for adherence but not at all for safer sex. Moreover, the larger significance of this pilot study is not well developed, as the connection to a randomized trial and subsequent dissemination and implementation is not clearly made.

2. Investigator(s):
Strengths
- The applicant has some expertise already and will clearly be dedicated and motivated to succeed in her first NIH grant as a PI.
- The co-investigators are highly accomplished in adherence to HIV therapies (as well as conducting qualitative research, pilot research, and RCTs) and apparently dedicated to the success of Dr. Whiteley.

Weaknesses
- The application seems to feature the abilities and achievements of the co-investigators over the application. The achievements of Dr. Whiteley relative to the proposed study activities do not appear to be included in the application.

3. Innovation:

Strengths

- Gaming is clearly an innovative idea and the use of “Viral Combat” as the starting point for a low-cost developmental process is an excellent idea.
- Linking the game via smartphone with the Wisepill device is extremely innovative.

Weaknesses

- The application does not adequately describe innovative methodology for the trial of 50 MSM. For example, it does not appear that any of the assessment occurs remotely even though all men will be issued a smartphone.

4. Approach:

Strengths

- Retention rates of the applicant and her co-investigators are impressive. It will be vital to use those same methodologies in the proposed study. Recruitment rates are also impressive.
- The use of “viral combat” as the starting point for the adaptation is very strong.
- The 1-month Wisepill only time is a strong point of the open trial design.
- The assessment schedule is a strong point, especially with the addition of the biological measures. The assessment of intracellular TFV-DP and FTC-TP using dried blood spots is extremely valuable to the study (although planned frequency of these assessments was not specifically included).

Weaknesses

- Whether MSM most at-risk of HIV and being prescribed PrEP will have the stability needed to store, carry, and thus use the electronic pill dispenser is not a question that was clearly entertained in the narrative. This very basic question is particularly important for MSM who are concealing their use of PrEP and for those not having a personal living environment. The insufficient testing this question is possibly an oversight of the proposed research project.
- The application does not seem to provide the number of MSM currently taking PrEP, who are also not enrolled in a PrEP-related study, in the recruitment site. This can be problematic as recruiting 90 MSM who are taking PrEP, but not enrolled in another PrEP-related study (at an institution billing itself as a leader in PrEP research) is not an automatic “win” – assurance that this recruitment goal can be easily met does not appear to be included.
- The COMP condition game (The Body) is not fully described, leaving open questions about what the intervention group is being compared to in this preliminary study. The use of the game by friends or sex partners of the intervention men does not seem to be offered as a possibility – this might be an oversight as contamination is entirely foreseeable.
- The application states: “Successful games are intuitive, engaging and inherently rewarding through action and feedback. Thus, many of the attributes of a successful game are a natural fit for a successful IMB-informed intervention” (p. 46). This logic connecting these two statements is not at all clear. It is not intuitive that I, M and B constructs will all be met through action and feedback.
- The appeal of Viral Combat to MSM is not fully described. The popularity of this game may be among heterosexuals. This is an unexplored possibility that may require extensive changes for MSM.
- Analysis plans for the trial of 50 MSM do not adequately describe how the two stratification variables will be handled. These two stratification variables will create very small cells yet dealing with that issue is not adequately described.
- The measure of HIV/STD knowledge is quite generic thus it seems odd to expect a change in this construct as a consequence of the gaming. It seems that a more PrEP-specific measure of knowledge might be far more suitable to the proposed study. The 14-item measure of PrEP taking skills suggests that these skills will be acquired through the gaming, yet the description of the gaming does not appear to include skill acquisition.

- The concept of detecting a .80 effect size does not seem feasible. This is a very large effect for an untested game. It is unclear why the application attempts to engage in significance testing with such small numbers (simple measures of effect size would likely be warranted).

5. Environment:
Strengths
- The Miriam Hospital, and Lifespan Hospital clearly offer outstanding environments for conducting the proposed research.

Weaknesses
- [None noted]

Protections for Human Subjects:
Acceptable Risks and/or Adequate Protections
Data and Safety Monitoring Plan (Applicable for Clinical Trials Only):
- Acceptable

Inclusion of Women, Minorities and Children and not IRB Exemption #4.
- Sex/Gender: Distribution justified scientifically
- Race/Ethnicity: Distribution justified scientifically
- Inclusion/Exclusion of Children under 21: Including ages < 21 justified scientifically
- The inclusion of young Black and Latino MSM is entirely justified given the scope of the current HIV epidemic in the U.S.

Resource Sharing Plans:
- Unacceptable
- This was not included in the application

Budget and Period of Support:
Recommend as Requested

THE FOLLOWING RESUME SECTIONS WERE PREPARED BY THE SCIENTIFIC REVIEW OFFICER TO SUMMARIZE THE OUTCOME OF DISCUSSIONS OF THE REVIEW COMMITTEE ON THE FOLLOWING ISSUES:

PROTECTION OF HUMAN SUBJECTS: ACCEPTABLE. Adequate protections are in place.

INCLUSION OF WOMEN PLAN: ACCEPTABLE. The distribution is justified scientifically; this study is only intended to focus on young MSM.

INCLUSION OF MINORITIES PLAN: ACCEPTABLE. The distribution minority and non-minority participates is scientifically justified.

INCLUSION OF CHILDREN PLAN: ACCEPTABLE. Children are included in this study at a justified scientific level.

COMMITTEE BUDGET RECOMMENDATIONS: The budget was recommended as requested.

ADMINISTRATIVE NOTE: It was noted during the review of the application that the appendix is inconsistent with a current notice on appropriate content for appendices (see NOT-OD-07-018). Specifically, the Appendix 1 (“Preliminary story board/ screen shots for Viral Combat”) includes information necessary to evaluate the Research Plan and would seem appropriate to include in that

section. In addition, it was noted that Internet web addresses (URLs) are included in the application. The inclusion of URLs is not allowed as indicated in the PHS SF424 (R&R) Application Guide. As stated in NOT-OD-11-080, “NIH has the authority to withdraw an application from review and funding consideration for egregious cases in which the Appendix or certain sections of the applications are used to circumvent page limits or include inappropriate material in the application.”

NIH has modified its policy regarding the receipt of resubmissions (amended applications). See Guide Notice NOT-OD-14-074 at http://grants.nih.gov/grants/guide/notice-files/NOT-OD-14-074.html. The impact/priority score is calculated after discussion of an application by averaging the overall scores (1-9) given by all voting reviewers on the committee and multiplying by 10. The criterion scores are submitted prior to the meeting by the individual reviewers assigned to an application, and are not discussed specifically at the review meeting or calculated into the overall impact score. Some applications also receive a percentile ranking. For details on the review process, see http://grants.nih.gov/grants/peer_review_process.htm#scoring.

MEETING ROSTER

National Institute of Mental Health Special Emphasis Panel
NATIONAL INSTITUTE OF MENTAL HEALTH
NIMH HIV/AIDS Review
ZMH1 ERB-K (07) V
July 10, 2014

CHAIRPERSON

LESCANO, CELIA M., PHD
RESEARCH ASSOCIATE PROFESSOR
DEPARTMENT OF MENTAL HEALTH LAW & POLICY
UNIVERSITY OF SOUTH FLORIDA
TAMPA, FL 33612

MEMBERS

BAPTISTE, DONNA R, EDD
SENIOR FACULTY & STAFF PSYCHOLOGIST
THE FAMILY INSTITUTE AT NORTHWESTERN UNIVERSITY
EVANSTON, IL 60201

BASSETT, INGRID VALERIE, MPH, MD
ASSOCIATE PROFESSOR
DEPARTMENT OF MEDICINE
MASSACHUSETTS GENERAL HOSPITAL
BOSTON, MA 02114

BUTLER, LISA MICHELLE, MPH, PHD
ASSOCIATE SCIENTIFIC RESEARCHER
DEPARTMENT OF GENERAL PEDIATRICS
BOSTON CHILDREN'S HOSPITAL
BOSTON, MA 02114

CROSBY, RICHARD A, PHD
ASSOCIATE PROFESSOR
COLLEGE OF PUBLIC HEALTH
UNIVERSITY OF KENTUCKY
LEXINGTON, KY 40506

HIGHTOW-WEIDMAN, LISA B, MPH, MD
CLINICAL ASSOCIATE PROFESSOR
DEPARTMENT OF MEDICINE
DIVISION OF INFECTIOUS DISEASES
UNIVERSITY OF NORTH CAROLINA SCHOOL OF MEDICINE
CHAPEL HILL, NC 27599

JONES, RACHEL , PHD
ASSOCIATE PROFESSOR
SCHOOL OF NURSING
NORTHEASTERN UNIVERSITY
BOSTON, MA 02115

KEMPF, MIRJAM-COLETTE , MPH, PHD
ASSOCIATE PROFESSOR
DEPARTMENT OF FAMILY/CHILD HEALTH & CAREGIVING
UNIVERSITY OF ALABAMA AT BIRMINGHAM
BIRMINGHAM , AL 35294

KENNEDY, STEPHEN B., MPH, MD
RESEARCH SCIENTIST
PACIFIC INSTITUTE FOR RESEARCH AND EVALUATION
LOUISVILLE CENTER
LOUISVILLE, KY 40208

KERSHAW, TRACE S, PHD
ASSOCIATE PROFESSOR
CENTER FOR INTERDISCIPLINARY RESEARCH ON AIDS
YALE UNIVERSITY
NEW HAVEN, CT 06510

KIPKE, MICHELE D, PHD
PROFESSOR
DEPARTMENT OF PEDIATRICS
KECK SCHOOL OF MEDICINE
UNIVERSITY OF SOUTHERN CALIFORNIA
LOS ANGELES, CA 90028

MILLER, ANDREW H, MD
WILLIAM P. TIMMIE PROFESSOR
DEPARTMENT OF PSYCHIATRY AND BEHAVIORAL SCIENCES
SCHOOL OF MEDICINE
EMORY UNIVERSITY
ATLANTA, GA 30322

PINKERTON, STEVEN D., PHD
PROFESSOR
DEPARTMENT OF PSYCHIATRY AND BEHAVIORAL MEDICINE
MEDICAL COLLEGE OF WISCONSIN
MILWAUKEE, WI 53202

RHODES, SCOTT D, MPH, PHD
PROFESSOR
DEPARTMENT OF SOCIAL SCIENCES AND HEALTH POLICY
WAKE FOREST UNIVERSITY SCHOOL OF MEDICINE
WINSTON-SALEM, NC 27157

VALOIS, ROBERT F., MPH, PHD
PROFESSOR
DEPARTMENT OF HEALTH PROMOTION, EDUCATION AND BEHAVIOR
ARNOLD SCHOOL OF PUBLIC HEALTH
UNIVERSITY OF SOUTH CAROLINA
COLUMBIA, SC 29208

VANABLE, PETER A., PHD
PROFESSOR AND CHAIR
DEPARTMENT OF PSYCHOLOGY
SYRACUSE UNIVERSITY
SYRACUSE, NY 13244

WEI, CHONGYI , DRPH
ASSISTANT PROFESSOR
EPIDEMIOLOGY & BIOSTATISTICS
UCSF SCHOOL OF MEDICINE
SAN FRANCISCO, CA 94143

WEINE, STEVAN MERRILL, MD
PROFESSOR
DEPARTMENT OF PSYCHIATRY
UNIVERSITY OF ILLINOIS
CHICAGO, IL 60612

WONG, FRANK Y., PHD
ASSOCIATE PROFESSOR
DEPARTMENT OF BEHAVIORAL SCIENCES
AND HEALTH EDUCATION
ROLLINS SCHOOL OF PUBLIC HEALTH
EMORY UNIVERSITY
ATLANTA, GA 30322

SCIENTIFIC REVIEW ADMINISTRATOR

GAVIN-EVANS, KAREN , PHD
SCIENTIFIC REVIEW OFFICER
DIVISION OF EXTRAMURAL ACTIVITIES
NATIONAL INSTITUTE OF MENTAL HEALTH
NATIONAL INSTITUTES OF HEALTH
BETHESDA, MD 20892

GRANTS TECHNICAL ASSISTANT

MUNDELL, TERRY
EXTRAMURAL SUPPORT ASSISTANT
DIVISION OF EXTRAMURAL ACTIVITIES
NATIONAL INSTITUTE OF MENTAL HEALTH
NATIONAL INSTITUTES OF HEALTH
BETHESDA, MD 20892

Consultants are required to absent themselves from the room during the review of any application if their presence would constitute or appear to constitute a conflict of interest.
